# Peer Group Identification as Determinant of Youth Behavior and the Role of Perceived Social Support in Problem Gambling

**DOI:** 10.1007/s10899-018-9813-8

**Published:** 2018-11-21

**Authors:** Iina Savolainen, Anu Sirola, Markus Kaakinen, Atte Oksanen

**Affiliations:** Faculty of Social Sciences, University of Tampere, 33100 Tampere, Finland

**Keywords:** Social identification, Social support, Problem gambling behavior, Youth

## Abstract

Gambling opportunities have increased rapidly during recent years. Previous research shows that gambling is a popular activity among youth, which may contribute to problem gambling. This study examined how social identification with online and offline peer groups associates with youth problem gambling behavior and if perceived social support buffers this relationship. Data were gathered with an online survey with 1212 American and 1200 Finnish participants between 15 and 25 years of age. Measures included the South Oaks Gambling Screen for problem gambling, and items for peer group identification and perceived social support. It was found that youth who identify strongly with offline peer groups were less likely to engage in problem gambling, while strong identification with online peer groups had the opposite effect. We also found that the associations between social identification and problem gambling behavior were moderated by perceived social support. Online peer groups may be a determinant in youth problem gambling. Focusing on offline peer groups and increasing social support can hold significant potential in youth gambling prevention.

## Introduction

Over the past decade, gambling has increased its popularity as a recreational activity (Molinaro et al. 2018; Orford 2010), particularly among individuals between 11 and 25 years of age, or, youth (Calado et al. 2017; UNESCO 2017). Even though gambling is illegal for underaged youth, new gambling technologies have made different forms of gambling widespread and much easier for even the youngest individuals to access (Blinn-Pike et al. 2010; Canale et al. 2016; Elton-Marshall et al. 2016). Above all, Internet gambling has transformed the traditional gambling landscape by offering convenient, instant, and constant access to novel gambling forms (Gainsbury et al. 2015; Griffiths and Parke 2010). Among a sample of ≥ 18-year-old college student problem gamblers, Petry and Gonzalez-Ibanez (2015) found that nearly half (49%) had gambled on the Internet during the month prior to the study. The sample included only those students who had scored more than 3 points on a combined gambling measure, consisting of the South Oaks Gambling Screen (SOGS) and money spent on gambling during the past 2 months. More recently, Molinaro et al. (2018) reported that 22.6% of 16-year-old students had gambled during the past year, 16.2% of them online.

Past research further indicates that the prevalence rate of gambling engagement is considerably high among youth and predominantly focused on private betting on skill-based games (Elton-Marshall et al. 2016; Volberg et al. 2010). An extensive review study from the turn of the decade found that, compared to 11% of adults, 28% of youth reported having bet on games of skill, such as card games, in the past year (Volberg et al. 2010). A more recent review consisting of 44 studies on gambling among 11- to 24-year-olds, concluded that up to 12.3% of youth within that age range qualify as problem gamblers across five continents (Calado et al. 2017).

Gambling activities can provide individuals with many subjective benefits, such as excitement, entertainment, and a perceived sense of acquiring wealth without much effort (Derevensky and Gilbeau 2015; Kim et al. 2017). However, both recreational and problematic gambling alike are associated with several psychosocial, physical, and mental health problems (Fröberg et al. 2015; Kong et al. 2013). Past research has found associations between gambling engagement and substance abuse (Calado et al. 2017; Kessler et al. 2008), increased financial difficulties (Raisamo et al. 2013), and poor school performance, as well as damaged social relationships (Raisamo et al. 2013; Splevins et al. 2010). Problem gambling is a growing global issue that may further manifest in a range of mental health problems, such as depression, anxiety, mood difficulties, and aggression (Lloyd et al. 2010; Yip et al. 2011). As these issues can become increasingly prevalent when the onset of gambling occurs prior to adulthood (Kong et al. 2013), new research perspectives are needed to explain youth gambling behavior and motives, as well as other underlying mechanisms.

## Social Identity as a Determinant of Behavior

Social relationships are recognized as a key determinant of overall well-being (Baumeister and Leary 1995; Thoits 2011) and behavior (Cruwys et al. 2015; Holt-Lunstad 2010) for people in general, but especially for young individuals in the 15–25 age group (Best et al. 2014a; Dishion and Tipsord 2011; Tarrant 2002). One possible linkage between social relationships and subsequent behavior is social identification, which is operationalized as the subjective sense of belonging to a certain group (Buckingham et al. 2013; Cruwys et al. 2017; Jetten et al. 2014). Social identification, as introduced by Tajfel and Turner (1979), refers to a process in which an individual’s identity is partly determined by his or her connectedness to desired social groups. These “in-groups” provide individuals with a sense of belonging and purpose, which have been shown to have significant outcomes in terms of personal capital and guide behavioral choices beyond normative peer influence (Best et al. 2014b; Frings and Albery 2015; Mawson et al. 2015).

According to the social identity theory (SIT), becoming a member of an “in-group” consisting of peers or similar others is beneficial for an individual (Tajfel and Turner 1979), as it enhances self-esteem, positive self-concept, and contributes to decision-making processes (Buckingham et al. 2013). Social psychological research has consistently recognized the impact that social identities have in shaping not only individuals’ beliefs, but various behaviors as well, ranging from positive health promotion to negative health-destructive behaviors (Jetten et al. 2012, 2014; Oyserman et al. 2007). Once social identity with a desired group is established, the individual is more motivated to behave in accordance with the groups’ perceived norms (Turner 1991; Marino et al. 2016). These social identity effects may be even more pronounced among youth who are still constructing their identities (Becht et al. 2017).

Kobus (2003) found that adolescents between ages 11 and 20 were more likely to engage in smoking behavior when their peer group identity was salient. Oyserman et al. (2007) concluded that, across seven experiments, race-related social identities were associated with either health-promotion or unhealthy behavior, depending on the social group with which the participants identified. Congruently, health-promoting behaviors were associated with belonging to the white and middle-class in-group identity, while unhealthy behaviors were associated with racial minority identities. A study by Foster et al. (2014) on college student gambling found that gambling behavior was associated with a stronger social identity with other gambling students, rather than the student body in general. Social identity was also found to moderate the association between perceived college norms and gambling behavior, as gambling students were more likely to perceive it as a normative behavior among their peers (Foster et al. 2014).

Peer relationships have a heightened importance among adolescents and young adults, and most adolescents report that they belong to a peer group (Chow et al. 2011; Flynn et al. 2017; Tarrant 2002). Due to the changing structure of the social world, individuals can now find meaningful groups with which to identify in both offline (Sussman et al. 2007) and online (Davis 2012; Mikal et al. 2016) environments. Ever since the introduction and exponential growth in popularity of the first social networking services, such as MySpace, Bebo, and Facebook—and, later on, Instagram, Twitter, and Snapchat—people have been connecting with ever-widening circles of other users and creating associations with individuals around the globe (Livingstone 2008; Tsitsika et al. 2014; Young 2011).

Social media is particularly attractive to young users: About 90% of young adults in the United States use social media platforms and more than half of them visit the sites daily (Villanti et al. 2017; Lin et al. 2016). Research has found that social media services are used mainly for communication with friends from the past and present (Davis 2012; Neira et al. 2014), sharing thoughts and interesting content (Kaplan and Haenlein 2010), and for self-disclosure and expression (Best et al. 2014a; Livingstone 2008). Research has also suggested that through online networks, individuals can more easily come in contact with like-minded others and share mutual ideas and content (Aiello et al. 2012; Sirola et al. 2018). Despite the fact that online and offline social networks tend to overlap, online social ties have been identified as an independent source of social connectedness that are not reducible to the ones originating from face-to-face interactions (Cole et al. 2017). Moreover, online social groups are valued as being equally important as those offline and building one’s identity through online venues and groups seems to be a growing modern norm (Borca et al. 2015; Lehdonvirta and Räsänen 2011).

Given its increasing popularity, youth gambling generates a new type of question in terms of social identities and the gambling phenomenon. Past research has been able to identify several individual, familial, and contextual factors associated with youth gambling (Buckle et al. 2013; Dussault et al. 2017), yet little research has examined whether social identity functions as an implicit mechanism reducing or inducing the behavior. It has been systematically reported that youth gambling and problem gambling are associated with the male gender (Splevins et al. 2010), young age, lower education level (Gainsbury et al. 2015; Hing et al. 2017), family-induced gambling experiences (Gupta and Derevensky 1997; Volberg et al. 2010) and having a positive attitude toward gambling (Dixon et al. 2016). These common features of gamblers and problem gamblers might suggest that gambling youth are socially motivated to either participate in the behavior or assimilate with a desired peer group via the behavior.

## Perceived Social Support

As discussed above, social identities’ impact on health and well-being is considerable. Equally important to health and well-being is the support derived from these social relationships (Cohen and Wills 1985; Dussault et al. 2016). These social mechanisms commonly go together, especially in psychology and health literature, where their impact is widely documented in patient and addiction recovery outcomes (Buckingham et al. 2013; Dingle et al. 2015a, b). For instance, Best et al. (2014a) found that rethinking identity processes allowed therapeutic community patients to recognize the social resources accessible for them while in alcohol and drug addiction. In the study, the patients explored potential transitional identities from “user” to “therapeutic community member.” Through this rethinking practice, the patients were better able to gain a sense of belonging and support. These were later associated with improved health outcomes, such as life-satisfaction and abstinence from drinking at follow-up (Best et al. 2014a).

In another study, Wu et al. (2016) found that social support was significantly associated with lower levels of Internet addiction among adolescents. These results were further supported by a meta-analysis showing evidence that adolescents and young adults with low support are at a higher risk of becoming addicted to Internet use (Lei et al. 2018). In terms of gambling behavior, research suggests that lack of perceived social support is a risk factor in youth gambling. One study found that youth who were at-risk or probable pathological gamblers also reported that they felt a lack of social support (Hardoon et al. 2004). Similarly, Petry and Weiss (2009) concluded that social support received from family members and friends significantly mediated both short-term and long-term gambling outcomes among pathological gamblers.

Previous research on perceived social support as a resource for recovery has further found that young individuals estimate their total personal, social, and recovery capital to be lower than that of older individuals, suggesting that these resources continue to strengthen over time and in conjunction with stronger social identities (Mawson et al. 2015). Given that social support received from meaningful groups can have significant outcomes in terms of decision making and the consequences that follow, youth are a particularly vulnerable group to the effects of perceived low social support. To this effect, earlier research findings indicate that strong social belonging and support can buffer youths against harmful online experiences (Kaakinen et al. 2018; Minkkinen et al. 2016). Notably, it was only the offline social relations that were found to have a buffering effect, while online social relations did not. In line with this finding, offline and online social connections have been reported to correlate with online risk behavior in inverse ways; while social ties offline are linked to a decreased likelihood of risk behavior, online relationships seem to have a reverse, increasing effect (Kaakinen et al. 2018). Considering the amplitude of harmful content and risky groups to which youth are exposed to online every day, investigating young individuals’ perceived social support capital is important and has extensive implications.

Social identities, in a form of meaningful group memberships and social support, are both social psychological explanations as to why social ties are important for human well-being (Jetten et al. 2014). More specifically, it has been suggested that social identities might contribute to well-being by making social support more accessible to individuals (Haslam et al. 2005). There is, however, a gap in research literature that assesses whether the positive outcomes of social identification are actualized when there is a lack of perceived social support. This would further guide our understanding of social identity dynamics and well-being.

## The Current Study

In the current study, youth between the ages of 15 and 25 are the population of interest and investigation, as these years include distinct developmental periods characterized by identity uncertainty and exploration (Archer 1982; Mawson et al. 2015). During these times, youth experiment with the diverse identities salient to them via different personal and social contexts online and offline. Using social identity theory as a theoretical framework, this cross-cultural study seeks to provide a supplementary explanation to youth problem gambling.

The aim of this research is to investigate and compare how social identification with peer groups online and offline is associated with problem gambling behavior among American and Finnish youths. More specifically, this research seeks to determine whether social identification is related to higher social support and if this relationship may safeguard youth from engaging in problem gambling, as theory indicates. While the beneficial outcomes of social identification and social support are widely reported (Haslam et al. 2005; Jetten et al. 2014), more research is needed on whether social identity is related to reduced destructive behavior (i.e., problem gambling) if perceived social support is absent.

The two countries were chosen because they are both technologically advanced Western countries while culturally diverse. Within both the United States and Finland, youth engage in gambling despite the existing gambling laws that aim to restrict such activity among underage individuals (i.e., those under 18 years of age in Finland and those under 21 years of age in several states of the United States). Cross-cultural research can provide deeper insight in understanding youth gambling. It is also needed to establish whether these social psychological phenomena exist in different cultural contexts. To our knowledge, no previous research has examined whether social identities of youth steer problem gambling behavior and if this possible effect is consistent across developed Western countries. Consequently, this study aims to contribute to the existing body of research by focusing on inspecting online and offline social identities as pathways to problem gambling behavior among youth, as well as by further examining whether perceived social support can moderate this connection.

Within both samples, we hypothesized that strong identification with an offline peer group is associated with lower engagement in problem gambling behavior (H1), while strong identification with an online peer group is associated with higher engagement in problem gambling behavior (H2). It was expected that high perceived social support is associated with less problem gambling behavior (H3). It was further hypothesized that perceived social support moderates the association between social identification and problem gambling behavior (H4).

## Method

### Participants

Participants were recruited from a volunteer pool administered by Survey Sampling International (SSI). The samples consisted of a total of 1212 American participants aged 15–25 (*M* = 20.05, *SD* = 3.19, 50.17% female) and 1200 Finnish young people aged 15–25 (*M* = 21.29, *SD* = 2.85, 50.00% female). Both samples were demographically balanced in terms of age, gender, and living area. The Academic Ethics Committee of the Tampere Region approved the research before implementation. Participation in the study was fully voluntary and all respondents were informed of the aims of the study prior to participation. The participants were aware that they could withdraw from the study at any time. Participation in the study did not inflict any harm on the participants.

#### Survey

Data were collected with the *YouGamble* online survey conducted from March to April 2017 in Finland and in January 2018 in the United States. Both datasets were collected with LimeSurvey software by using identical survey formats. The surveys were optimized for both computers and mobile devices. The average survey response time was 14 min and 49 s in the United States and 15 min 30 s in Finland. The original survey was in Finnish. It was translated into English and back-translated again to ensure consistency and accurate matching of the survey items. Some questions were slightly modified to better fit the cultural setting in each country. The surveys were fully anonymous and included measures for all target variables, including gambling behavior, perceived social support, and identification with a primary peer group.

### Measures

The South Oaks Gambling Screen (SOGS) was used to measure the frequency and intensity of problem gambling behavior. The SOGS is regularly used in studies to screen for pathological gambling behavior (Lesieur and Blume 1987; Salonen et al. 2017). Some of the test items were slightly modified to accommodate for cultural variations in gambling. The scale had good internal consistency in both the American (α = .90) and Finnish (α = .89) sample. The items were summed up to a continuous scale measuring the level of engagement in problem gambling behavior. Earlier studies have identified problems with the SOGS cut-off scores which may lead to biased estimates of gambling problems and high rates of false positives (Battersby et al. 2002; Stinchfield 2002). To account for this, the SOGS was used as a continuous measure in our analyses, measuring the intensity of problem gambling behavior, instead of categorizing respondents to problem gamblers. Using the SOGS measure as a continuous variable also responds to the methodological criticisms raised from categorizing continuous outcome variables (Altman 2014). The suggested SOGS cut-off scores; 0–2 = no problem gambling, 3–7 = at-risk gambling, and ≥ 8 = probable pathological gambling, were used to provide descriptive statistics (Goodie et al. 2013).

Identification with a primary peer group consisting of friends or an online community was assessed with a survey item that inquired about the subjective sense of belonging to a primary peer group. The item was written as: “How strongly do you feel you belong to the following?” The group-type options provided for this inquiry included “a friendship group” and “an online community.” Answers could be provided on a scale ranging from 1 (*no belonging at all*) to 10 (*very strong belonging*).

Perceived social support was assessed with a survey item asking about the support an individual receives from close ones. The item asked: “Do you feel you receive support from your close ones when you need it?” Three answer choices were provided for the item: 1 = *Rarely*, 2 = *Sometimes*, 3 = *Often*. This question was then turned into a dummy variable (0 = *rarely*; 1 = *sometimes or often*).

### Statistical Analysis

Descriptive statistics for all continuous variables were calculated as means (*M*) and standard deviations (*SD*), and as frequencies (*n*) and relational proportions (%) for categorical variables. This information is presented in detail in Table 1. In order to compare differences between the two countries and statistically test the hypotheses, linear regression analysis was conducted. Two separate regression models were conducted for both countries to analyze the direct effects of social identification and support on problem gambling behavior (see Table 2).

**Table 1:** Descriptive statistics. Continuous variables reported as means (M) and standard deviations (SD), categorical variables as frequencies (n) and relational proportions (%)

Variable	United States			Finland		
	*M*	*SD*	Range	*M*	*SD*	Range
Problem gambling	1.27	2.55	0–20	1.59	2.56	0–20
Identification w/offline peers	6.72	2.62	1–10	6.83	2.49	1–10
Identification w/online peers	5.38	2.69	1–10	5.04	2.61	1–10
Age	20.05	3.19	15–25	21.29	2.85	15–25

Categorical variables	Coding	*n*	%	Coding	*n*	%
Gender	Male	604	49.83	Male	600	50
	Female	608	50.17	Female	600	50
Perceived social support	Weak	212	17.49	Weak	112	9.33
	Strong	1000	82.51	strong	1088	90.67
SOGS cut-off score	0–2	1011	83.42	0–2	946	78.83
	3–7	157	12.95	3–7	210	17.50
	≥ 8	44	3.63	≥ 8	44	3.67

The South Oaks Gambling Screen (SOGS) cut-off scores used were: *no problem gambling* (0–2), *at risk gambling* (3–7) and *probable pathological gambling* (≥ 8)

**Table 2:** Main effects of the model predicting problem gambling in the United States (N = 1212) and Finland (N = 1200)

Variable	US				Finland			
	*B*	*SE*	*p*	*β*	*B*	*SE*	*p*	*β*
Identifying w/offline peers	**− 0.07**	**0.03**	**0.034**	**− 0.07**	**− 0.11**	**0.03**	**0.001**	**− 0.11**
Identifying w/online peers	**0.17**	**0.03**	**0.000**	**0.17**	0.01	0.03	0.868	0.01
Perceived social support	**− 0.71**	**0.20**	**0.000**	**− 0.10**	− 0.10	0.26	0.709	− 0.011
Age	**0.16**	**0.02**	**0.000**	**0.19**	0.01	0.03	0.718	0.01
Gender	**− 0.75**	**0.15**	**0.000**	**− 0.14**	**− 1.22**	**0.15**	**0.000**	**− 0.24**

Statistically significant results (*p* < .05) boldfaced. Gender was represented as a dummy variable with code 1 serving as the male reference group. Perceived social support measured as a dummy variable with code 1 serving as the “strong support” reference group

Interaction analysis was conducted with regression analysis while treating perceived social support as a moderator. This approach was also applied separately for both countries to allow for better observation of the ways in which the effects vary by a given country. These results are reported in Table 3. We conducted the moderation analysis by first testing the statistical significances of both interaction terms in the regression models. Secondly, the slope difference analysis suggested by Robinson et al. (2013) was used to analyze a difference in the independent variables’ regression coefficients over the values of the dichotomous moderator variable. The slope difference test is less conservative than interaction term testing and, thus, is less prone to Type II errors (failing to reject a false null hypothesis). In the interaction analysis, social identification variables were mean-centered in both samples to avoid multicollinearity. Gender and age were treated as controls in all models.

**Table 3:** Regression results of the moderation analyses for problem gambling in the United States (N = 1212) and Finland (N = 1200)

Variable	US				Finland			
	*B*	*SE*	*p*	*β*	*B*	*SE*	*p*	*β*
Identification with offline peers	0.05	0.07	0.523	0.05	0.08	0.09	0.408	0.07
Identification with online peers	**0.17**	**0.07**	**0.015**	**0.17**	**0.25**	**0.10**	**0.011**	**0.26**
Perceived social support	**− 0.86**	**0.22**	**0.000**	− **0.12**	**2.19**	**0.56**	**0.000**	**0.25**
Perceived social support × identific w/offline peers	− 0.15	0.08	0.078	− 0.12	− **0.22**	**0.10**	**0.024**	− **0.26**
Perceived social support × identific w/online peers	0.00	0.08	0.984	0.00	− **0.27**	**0.10**	**0.009**	− **0.31**
Age	**0.15**	**0.02**	**0.000**	**0.18**	0.01	0.03	0.794	0.01
Gender	− **0.75**	**0.15**	**0.000**	− **0.14**	− **1.18**	**0.15**	**0.000**	− **0.23**

Statistically significant results (*p* < .05) boldfaced. Gender was represented as a dummy variable with code 1 serving as the male reference group. Perceived social support measured as a dummy variable with code 1 serving as the “strong support” reference group

## Results

According to our models, identification with a primary offline peer group had a significant association with lower problem gambling behavior (see Table 2). This was true both in the United States (*B* = − .07, *SE* = .03, *t*(1211) = − 2.12, *p* = .034) and in Finland (*B* = − .11, *SE* = .03, *t*(1199) = − 3.34, *p* = .001). Identifying with a primary online peer group was associated with higher engagement in problem gambling behavior, but the effect was significant only among U.S. youths (*B *= .17, *SE* = .03, *t*(1211) = 5.68, *p* < .001). This finding supports our hypothesis that strong identification with online peers is associated with higher gambling behavior. High perceived social support was associated with lower problem gambling behavior, but this direct effect was also significant only among the U.S. sample (*B* = − .71 *SE* = .20, *t*(1211) = − 3.53, *p* < .001). Of our covariates, only gender was associated with engaging in problem gambling behavior in both countries, as male respondents reported significantly higher rates of problem gambling behavior than females in the U.S. (*B* = − .75, *t*(1211) = − 5.8, *p* < .001) and in Finland (*B* = − 1.22, *SE* = .15, *t*(1199) = − 8.13, *p* < .001). Age, in turn, was not associated with problem gambling behavior in Finland (*B* = .01, *SE* = .03, *t*(1199) = .26, *p* = .718), but was associated with it in the United States (*B* = .16, SE = .02, *t*(1211) = 6.72, *p* < .001).

In terms of our moderation analysis (Table 3), it was found that high perceived social support significantly moderated the association between engagement in problem gambling behavior and peer group identification with both offline (*B* = − .22, *SE* = .10, *t*(1199) = − 2.27, *p* = .024) and online (*B* = − .27, *SE* = .10, *t*(1199) = − 2.61, *p* = .009) peers among Finnish youths. With the U.S. sample, the interaction term between high perceived social support, social identification, and problem gambling behavior was not significant in either offline (*B* = − .15, *SE* = .08, *t*(1211) = − 1.76, *p* = .078) or online (*B *= .00, *SE* = .08, *t*(1211) = − .02, *p* = .984) peer groups. According to the slope difference test, the slope for social identification with an offline peer group differed significantly between those who report perceived social support and those who do not (*F*(1, 1204) = 16.50, *p* < .001). In case of online identification, such a difference did not exist (*F*(1, 1204) = 0.00, *p* = .964). Notably, identification with offline peer groups was only associated with decreased problem gambling behavior if respondents also reported at least some degree of social support.

## Discussion

This study examined the effects of peer group identification on problem gambling behavior among American and Finnish youth. Our data suggested that both U.S. and Finnish youth identify with peers online and offline and engage in gambling activities to a similar degree. Our multivariate results, in turn, found varying but significant relationships between the examined variables within both samples. Social identification with an offline peer group was associated with lower levels of problem gambling behavior among both U.S. and Finnish youths. In the United States, identification with an online peer group was associated with higher levels of problem gambling behavior. Also, among U.S. youths, social support was associated with lower levels of problem gambling behavior.

In both the U.S. and Finnish samples, perceived social support moderated the association between problem gambling behavior and social identification offline, as the negative association existed only if respondents also reported perceived social support. In the Finnish sample, the moderation effect was found with online identification as well. In this case, online identification was associated with higher levels of problem gambling behavior among those who did not report perceived social support.

The results of the study mostly supported our hypotheses. Our findings also supported prior research on social identification with primary offline groups functioning as an element in determining youth behavior. The observed positive association between online identification and engagement in problem gambling behavior in the United States is consistent with earlier studies emphasizing that offline and online social ties may have different outcomes in terms of youth health and well-being (Kaakinen et al. 2018; Minkkinen et al. 2016). The association between perceived social support and lower levels of problem gambling behavior in the United States was also consistent with prior literature on social support theory, its effects and benefits: Those individuals who perceive they receive support when needed also report lower levels of problem gambling behavior.

According to our results regarding the moderation effect, it appears that the outcomes of social identification fluctuate as an effect of the support received from social relations. It is likely that there are qualitative differences in friend groups (e.g., a perceived normative stance toward gambling) and between those groups who report social support and those who do not, and these differences then may determine how social identity relates to addictive or otherwise harmful behaviors. This is in line with earlier research findings highlighting that identification may have different consequences on behavior depending on the groups with which one identifies (Foster et al. 2014; Mawson et al. 2015; Oyserman et al. 2007). The inverse results concerning online community identification in the United States probably reflect this same issue. In both the United States and Finland, offline peers seem to safeguard against problem gambling behavior when youth also report perceived social support. This could also be an indication that, with real-life peers, other types of activities are preferred, such as engaging in drinking or partying (Boman et al. 2013).

Further, in the United States, physical gambling venues are separated from other types of businesses and highly restricted from underaged individuals. This division might increase gambling’s enigmatic and “coolness” value in the eyes of youth and steer them toward gambling-related social contacts online, where others who share that interest can easily gather and meet. In the Finnish context, in turn, gambling can be performed with relative ease, even in real-life settings: slot machines, lottery, and scratch tickets are readily available at public venues, such as grocery stores and kiosks. It is not uncommon for groups of youths to gather around these venues and play when unobserved by the authorities. For youths who engage in gambling, the behavior might induce togetherness and a sense of shared experience. It is also possible that these youths possess social connections without the sense of perceived social support, which then fail to function as a protective factor. This social dynamic has been found to even increase gambling behavior, particularly among males (Yücel et al. 2015). Online identification was found to be a risk factor for problem gambling among those Finnish youth who did not receive social support from their social networks. Thus, it is further suggested that those youth with weak social ties offline might seek like-minded others online with whom they come to share gambling-related ideas and content (Aiello et al. 2012; Sirola et al. 2018).

Overall, our analyses showed that problem gambling behavior among youths might be, at least partially, identity-based and motivated. As gambling has become more popular among young people in recent years, it could be inferred that those youths interested in gambling are more motivated to seek and engage with online social networks that share their interest in gambling activities. A recent study examining family influence on gambling (Westberg et al. 2017), found that children and adolescents who witnessed gambling within the family, also perceived it as a part of their family identity. These children and adolescents were further found to normalize gambling behavior, not only in their immediate social surrounding, but in the general society as well, and consequently, engage in the behavior accordingly. Thus, it is conceivable that youth who explore and strengthen their identity through gambling peer groups might come to perceive the behavior as normative and desired (see also Foster et al. 2014) and thus engage in the activity in increasing volumes.

Consistent with previous literature suggesting that gambling can be considered a lonesome behavior (Edgren et al. 2016; Khazaal et al. 2017), we anticipated it to be carried out more frequently by individuals who do not have immediate primary groups with which to identify (Savolainen et al. 2018). Gambling is then used as a gateway to acceptance with, and integration into, peer groups. This might be particularly true with youths who take part in online communities, where they are exposed to vast amounts of behavior-specific content. Within these immediate online environments, specific behavioral content may quickly become the perceived norms and youth will begin to follow them. Consistent with the notion that similar-minded peers can be found online, our study found that those youths who reported belonging with mainly online peer groups, were also more likely to engage in problem gambling behavior.

This study has several implications that pertain to policy and practice. Instead of focusing on gambling on a one-person level, looking at the individual as a part of a social context and the support s/he has available can have significant outcomes in terms of preventing youth from engaging in problem gambling behavior. Given that much of youth gambling nowadays takes place online, as well as the research findings that the high use of social networking services is connected to poor psychological functioning among children and adolescents (Sampasa-Kanyinga and Lewis 2015), it can be predicted that spending excessive amounts of time in online gambling communities and sites induces similar negative outcomes in addition to the harm caused by problem gambling itself. Peer groups should be acknowledged as meaningful social contexts for youths. It is suggested that youths are better informed and educated about the importance of their social environments and the influences they may inflict. Shifting memberships from one peer group to another (e.g., from gambling groups to non-gambling groups) may be helpful in refraining from problematic behavior and reconstructing healthy social identities. Furthermore, the inclusion of social support means into prevention work could buffer young gamblers against problem gambling and decrease the amount of time used gambling.

### Limitations and Future Directions

While this study provides additional insight in existing gambling literature, some limitations must be acknowledged. The cross-sectional design utilized in this study does not allow for concluding that the examined associations have a causal relationship. Thus, it cannot be determined whether gambling behavior takes place prior to identification with peers, or if it is adapted through social identification mechanisms. Future research using longitudinal methods should thus examine whether or not the said causality exists. As this study did not scrutinize those with whom youth engage in the activity, future research should investigate whether it is carried out in isolation or in groups of other individuals.

Future research should also examine whether social support derived from family differs qualitatively from that received from peers, and if these have varying outcomes in terms of problem gambling prevention, given that this study did not separate the quality or nature of the social support received. Additional research is needed to investigate how online and offline peer group relationships differ qualitatively, functionally, and interpersonally, as they can have varying effects on youth behavior. This study anticipated that peer relationship type and their outcomes would differ across online and offline groups, yet it was not examined what underlying features drove these differences.

## Conclusion

This study examined the relationship between peer group identification and problem gambling behavior among young individuals in the United States and Finland. It was found that youth who identify strongly with offline peer groups are less likely to engage in problem gambling behavior, while identification with an online peer group had the opposite effect. Perceived social support moderates the association between peer group identification and problem gambling behavior. In both the U.S. and Finnish samples, offline identification was only associated with lower levels of problem gambling behavior if respondents also reported receiving social support. In the Finnish sample, problem gambling behavior was more prevalent among those youths who strongly identified with an online peer group but lacked social support. These results support previous research that youth behavior is partly determined by social identification but, here, social support was found to significantly moderate this mechanism.
